# Drugs or dissection? Unraveling a diagnostic puzzle in the ED: A case report on Tizanidine-Nifedipine interaction

**DOI:** 10.1016/j.toxrep.2025.102055

**Published:** 2025-05-27

**Authors:** Muhammad Abd Ur Rehman, Maaz Uddin Mohammed, Muhammad Abdul Mannan, Waleed Salem

**Affiliations:** a Clinical Fellow Medical Toxicology, Hamad Medical Corporation, Qatar; b Clinical Pharmacist, Emergency Department, Hamad Medical Corporation, Qatar; c Medical Intern, Liaquat University (Civil) Hospital, Hyderabad, Pakistan

**Keywords:** Calcium Channel Blockers, Pharmacodynamics, Drug-Drug Interaction, Synergism, Tizanidine, Central adrenergic receptor agonist, Nifedipine, Case Report

## Abstract

Lower back pain is among the top ten reasons patients visit the emergency department (ED). Tizanidine, a centrally acting alpha-2 adrenergic receptor agonist, is commonly prescribed for managing spasticity in patients with cerebral or spinal injuries. It is also used as an effective treatment for nonspecific lower back pain. One of the most critical and life-threatening causes of lower back pain is abdominal aortic dissection, particularly in patients with hypertension. Nifedipine, a 1,4-dihydropyridine calcium channel blocker (CCB), is a widely used oral antihypertensive and antianginal agent. It lowers systemic vascular resistance and dilates coronary arteries by inhibiting calcium ion (Ca²⁺) entry into the smooth muscle cells of small arteries (arterioles), thereby reducing systemic blood pressure and improving myocardial oxygen delivery. We report a compelling case of a male patient presenting to the ED with high blood pressure and lower back pain. Shortly after the administration of tizanidine and nifedipine, his blood pressure dropped significantly within an hour. Initially suspected to be a ruptured abdominal aortic dissection, the cause was later identified as a drug-drug interaction. The synergistic effects of tizanidine and nifedipine resulted in a rapid and critical drop in blood pressure. The Drug Interaction Probability Scale (DIPS) score of 4 suggests the hypotensive episode was possibly caused by a drug interaction. This case also highlights the importance of point of care ultrasound (POCUS) and repeated examinations in excluding life-threatening conditions like aortic rupture.

## Highlights

• First reported case of tizanidine–nifedipine interaction causing severe hypotension mimicking aortic dissection.
• Highlights need for caution when combining antihypertensives with alpha-2 agonists to avoid severe hypotension.
• Emphasizes a pharmacodynamic interaction, with possible pharmacokinetic involvement needing further study.

## 1 Introduction

Tizanidine is a fascinating medication that primarily targets central alpha-2 receptors, with additional effects mediated through imidazole receptors contributing to its actions. It works by inhibiting the presynaptic release of excitatory neurotransmitters and diminishing the effectiveness of these neurotransmitters on postsynaptic receptors [1]. This distinctive mechanism improves spasticity effectively without causing significant muscle weakness. In addition to its myotonolytic properties, tizanidine also lowers blood pressure, exhibits dose-dependent antinociceptive activity, provides sedative and anticonvulsant effects [2], inhibits gastrointestinal motility, and protects against drug-induced ulcers [3].

Nifedipine, a dihydropyridine calcium channel blocker (CCB), inhibits voltage-gated L-type calcium channels, reducing calcium (Ca²⁺) influx into cells and subsequently lowering intracellular Ca²⁺ levels. Since smooth muscle action potentials rely on calcium influx, nifedipine’s inhibition causes smooth muscle relaxation, resulting in vasodilation and a reduction in blood pressure [4]. As a dihydropyridine CCB, nifedipine selectively targets L-type calcium channels in vascular smooth muscle cells, with minimal affinity for these channels in cardiac pacemaker cells. This selectivity prevents nifedipine from causing bradycardia [5].

There have been multiple case reports documenting a marked decrease in blood pressure following the coadministration of tizanidine with angiotensin-converting enzyme inhibitors (ACE inhibitors) or angiotensin receptor blockers (ARBs), attributed to the synergistic effects of these drugs [6], [7], [8], [9]. Theoretically, a similar interaction could occur with calcium channel blockers (CCBs), but no such cases have been reported to date. To the best of our knowledge, this is the first reported case in medical literature highlighting a drug-drug interaction between tizanidine and nifedipine, resulting in a significant reduction in blood pressure within an hour of administration in the ED.

## 2 Case description

A 53-year-old male with a history of hypertension and diabetes presented with severe lower back pain that had persisted for two days. For more than two years he was not taking any medications for his hypertension and diabetes. The pain was continuous, and he reported no history of trauma, fever, vomiting, dysuria, fecal or urinary retention or incontinence.

Upon examination, the patient was alert but clearly in distress from the pain. Neurological assessment revealed motor strength of 5/5 and intact sensation in all extremities. There was notable tenderness in the lower midline, but no spinal deformities or evidence of radicular pain were found. Straight leg raise tests were negative, and a rectal examination showed normal tone with no saddle anesthesia.

Initial vital signs indicated significant hypertension, with a blood pressure (BP) of 193/130 mmHg with normal heart rate (82 beats/min). Blood pressure in the lower limb was 183/122 mmHg. Our 2 initial working diagnoses were lumbago or aortic dissection. We administered 1000 mg paracetamol and 30 mg ketorolac, intravenously for pain management. and did bedside Point-of-care ultrasound (POCUS) to look mainly for abdominal aorta. It showed normal sized abdominal aorta (1.3 cm and 1.7 cm at two different places) with no evidence of abdominal aortic aneurysm (AAA) or aortic dissection (AD). There was good cardiac contractility, no pericardial effusion, and no free fluid in the abdomen.

After 2 hours of his hospital stay, he started feeling nauseous and vomited once as well. We administered 4 mg of ondansetron intravenously. Considering his persistent high BP (188/120 mmHg after 2 hours), lower back pain with vomiting, we planned to do blood investigations including a computed tomography (CT) scan of his head to look for any end-organ damage, intracranial bleed or any space occupying lesion.

At this time, we administered 4 mg of oral tizanidine (after he had already received paracetamol and ketorolac) and 20 mg of sustained release formula of oral nifedipine for pain management and symptomatic hypertension, respectively. We chose nifedipine for its sustained-release, oral formulation, and—given the absence of any end-organ damage—deemed it the most effective and safest agent for lowering his blood pressure. After almost one hour of administration of these drugs, the patient started complaining of dizziness. When we assessed the patient, he was severely diaphoretic and was complaining of light-headedness. His BP was 111/71 mmHg. We immediately thought of the suspected aortic rupture. POCUS was repeated & again demonstrated no free fluid in abdomen, normal sized aorta, no evidence of AAA or AD, good cardiac contractility, with no pericardial or pleural effusion. Electrocardiograph (ECG) and the basic laboratory tests were unremarkable, other than raised d-dimer (0.89 mg/L FEU). He was constantly diaphoretic and was feeling light-headedness. Repeated BP reading was even lower (86/55 mmHg), but his heart rate was still within normal limits (77 beats/min). Random blood sugar was 5.7 mmol/L.

**Fig. 1:**
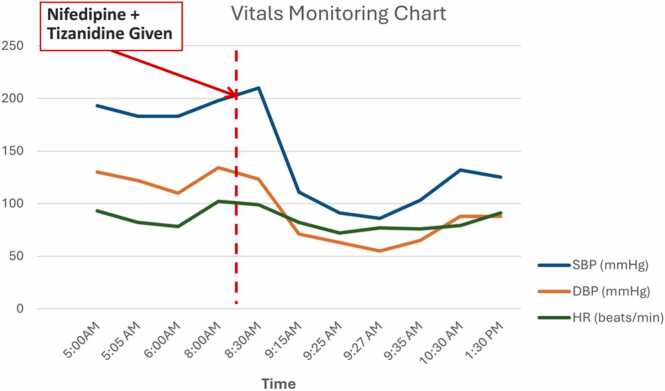
Graphical representation of the patient’s vital signs (before & after drug administration).

Considering this we placed him in a Trendelenburg position and administered intravenous fluid (IVF) bolus of one liter in 15 min and arranged an urgent ECG gated CT scan of the aorta along with CT scan of head to rule out aortic and intracranial pathologies, respectively. Another liter of IVF was given during the CT scan. After IVFs, his BP started showing improvement and rose to 103/65 mmHg. CT showed no signs of intracranial pathology and no evidence of aortic dissection, rupture, or aneurysm. The patient was kept under observation for the next four hours and showed a steady improvement in his vital signs as shown in Fig. 1.

Thinking retrospectively, we could not find any cause of his rapid deterioration with normal heart rate, other than possible synergistic effect of alpha-2 adrenoreceptor agonist (tizanidine) and calcium channel blockers (nifedipine). The patient was communicated about this unforeseen pharmacologic effect of the two drugs administered for different purposes. He was discharged home on symptomatic treatment for his lower back pain on non-steroidal anti-inflammatory drugs (NSAIDs) and acetaminophen. He was referred to the hypertension clinic where he was seen after almost two months and was not having any recurrence of these symptoms. However, during his clinic visit, he was prescribed a 5 mg daily dose of amlodipine for blood pressure management.

## 3 Discussion

According to the National Hospital Ambulatory Medical Care Survey (NHAMCS) conducted by the National Center for Health Statistics (NCHS), approximately 3.15 million patients visited emergency departments (EDs) annually in the United States with back symptoms between 2016 and 2022 [10]. In a prospective study involving twelve EDs across five countries, lumbar pain was the presenting complaint in 14.2 % of cases with acute aortic syndromes (AAS), and 72.2 % of these patients were hypertensive [11]. Given this context, our patient, with a history of hypertension, presenting to the ED with lower back pain and significantly elevated blood pressure, prioritizing the exclusion of AAS (including aortic dissection) was essential. After administering appropriate analgesia (acetaminophen and ketorolac), investigations were conducted, including bedside POCUS and d-dimer measurement. This approach aligns with the conservative and safe strategy recommended by the PROFUNDUS study, a multi-centered European study that demonstrated 100 % sensitivity in ruling out suspected AAS when combining bedside POCUS with d-dimer testing. This protocol reduced the need for advanced imaging in 41–54 % of patients, depending on the d-dimer cut-off applied [11].

Although recent recommendations have become stricter about treating asymptomatic markedly elevated blood pressure (formerly termed hypertensive urgency), the 2017 American Heart Association (AHA) guidelines still support using oral antihypertensive agents to manage high blood pressure in adults (>180/120 mmHg) without signs of end-organ damage [12], [13]. Accordingly, nifedipine was administered following these guidelines.

Nifedipine, developed by Bayer, was first introduced in the literature along with other dihydropyridines in 1972 [14]. As a dihydropyridine calcium channel blocker, it inhibits Ca²⁺ influx in smooth muscle cells, causing vasodilation and a subsequent reduction in blood pressure [4]. In 1998, Champlain et al. reported that nifedipine is associated with a transient increase in blood norepinephrine levels [15]. This elevation, combined with reflex tachycardia triggered by baroreceptor stimulation due to the drop in blood pressure, contributes to the reflex tachycardia observed after nifedipine administration [16]. Additionally, nifedipine has been shown to inhibit phenacetin O-deethylase activity (POD) by suppressing cytochrome P450 1A2 (CYP1A2) activity [17], [18]. However, it is primarily metabolized in the liver by CYP3A4 [18].

Tizanidine, a centrally acting alpha-2 adrenergic receptor agonist, is metabolized by CYP1A2. While studies have shown a modest reduction in diastolic blood pressure (12–15 %) following isolated tizanidine administration, its overall impact on blood pressure is minimal [19], [20]. However, when co-administered with drugs like ciprofloxacin or under conditions such as liver cirrhosis that inhibit CYP1A2 activity, significant hypotension can occur [21], [22]. Furthermore, tizanidine's inhibition of excitatory neurotransmitter release reduces heart rate and suppresses reflex tachycardia [1]. This effect can lead to pronounced hypotension when combined with antihypertensive drugs targeting the renin-angiotensin-aldosterone system (RAAS), such as ACE inhibitors (e.g., lisinopril) or ARBs (e.g., telmisartan) [6], [7], [8], [9]. Table 1 is summarizing the previously reported case of tizanidine interaction with different antihypertensive drugs and CYP1A2 inhibitors.

**Table 1:** Previously published articles on important tizanidine drug interactions.

	**Study Type**	**Age (years)**	**Interacting Drug**	**Complication**	**Mechanism**
**Publow et al.**[6]	Case report	85	Lisinopril	Hypotension, Bradycardia	Blockade of sympathetic response of hypotension
**Johnson et al.**[7]	Case report	10	Lisinopril	Hypotension	Blockade of sympathetic response of hypotension
**Kao et al.**[8]	Case report	48	Lisinopril	Hypotension	Blockade of sympathetic response of hypotension
**Mahajan et al.**[9]	Case report	56	Telmisartan	Hypotension, Bradycardia	Blockade of sympathetic response of hypotension
**Chaugai et al.**[21]	Retrospective Cohort Study	52 (median)^a^	Ciprofloxacin^b^	Hypotension	CYP1A2 inhibition

a As this was a retrospective cohort study, age is reported as the median.

b Ciprofloxacin and fluvoxamine were administered as CYP1A2 inhibitors in this study. However, we only mentioned ciprofloxacin because it was administered in 98.09 % of patients taking tizanidine.

**Fig. 2:**
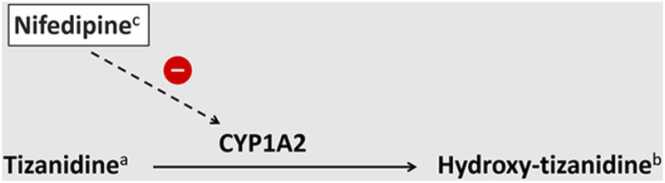
Showing inhibition of cytochrome P450 1A2 (CYP1A2) by nifedipine, resulting in slower metabolism of tizanidine. ^a^5-Chloro-N-(2-imidazolin-2-yl)-2,1,3-benzothiadiazol-4-amine. ^*b*^2-[(5-Chloro-2,1,3-benzothiadiazol-4-yl)amino]-4,5-dihydro-1H-imidazol-4-ol. ^*c*^Dimethyl 2,6-dimethyl-4-(2-nitrophenyl)-1,4-dihydropyridine-3,5-dicarboxylate.

**Fig. 3:**
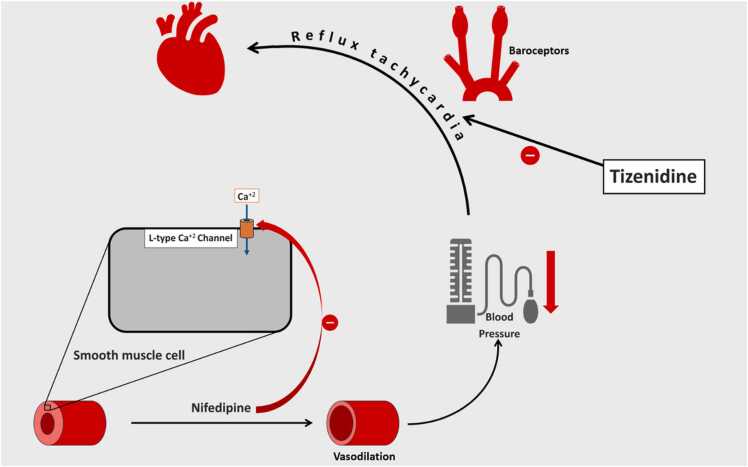
Showing mechanism of action of nifedipine and inhibition of reflex tachycardia by tizanidine, resulting in worsening of hypotension.

The interaction between nifedipine and tizanidine in our case followed a similar mechanism. Nifedipine inhibits CYP1A2, reducing tizanidine metabolism and prolonging its effects (Fig. 2), while tizanidine suppresses the reflex tachycardia typically triggered by the peripheral vasodilatory action of nifedipine (Fig. 3), exacerbating hypotension. We hypothesize that this combination led to the marked blood pressure decline observed within an hour of administration, with a nearly 50 % reduction from baseline. The hypotension responded effectively to intravenous fluid administration within the next hour.

We would like to highlight that the primary cause of hypotension in this case was pharmacodynamic in nature, similar to other antihypertensive drug interactions listed in Table 1 [6], [7], [8], [9]. The evidence for a pharmacokinetic interaction—specifically, the slower metabolism of tizanidine due to CYP1A2 inhibition—is relatively weaker. The only available supporting evidence comes from researches conducted over two decades ago. However, the study by Katoh et al. provides a thorough analysis of the pharmacokinetic effects of different CCBs in the context of CYP450 inhibition, suggesting that nifedipine is a strong competitive inhibitor of CYP1A2 [18]. Although we did not find additional studies confirming this interaction, no evidence contradicts it either. Therefore, the possibility of a pharmacokinetic interaction cannot be completely ruled out.

We also evaluated this interaction on The Drug Interaction Probability Scale (DIPS) which is a structured tool used to assess the likelihood that an observed clinical event is due to a drug-drug interaction. It consists of 10 weighted questions, each with specific scoring criteria. The final score determines whether the interaction is doubtful, possible, probable, or highly probable [23]. In this case, the DIPS score was 4, which suggests that the hypotensive episode was a “possible” drug interaction, but the evidence is not strong enough to confirm it definitively. While nifedipine and tizanidine’s synergistic pharmacodynamic effects make the interaction plausible, additional studies would be needed to establish a "probable" or "definite" classification.

## 4 Conclusion

In conclusion, tizanidine is generally a safe medication with therapeutic benefits, including muscle relaxation, analgesia, and protection against drug-induced ulcers. However, caution is advised when prescribing it to patients with liver impairment, in combination with antihypertensive drugs, or alongside CYP1A2 inhibitors due to the risk of significant hypotension. This case underscores the need for further research to definitively establish the impact of nifedipine on CYP1A2 as the evidence to this mechanism is weaker and we consider it as a limitation of the study. Additionally, it highlights the importance of POCUS and repeated clinical assessments in ensuring patient safety, particularly when ruling out life-threatening conditions such as aortic rupture.

## CRediT authorship contribution statement

**Muhammad Abd Ur Rehman:** Visualization, Conceptualization. **Waleed Salem:** Supervision. **Muhammad Abdul Mannan:** Writing – review & editing, Data curation. **Maaz Uddin Mohammed:** Writing – original draft, Validation.

## Author statement

Muhammad Abd Ur Rehman contributed to conceptualization and visualization; Maaz Uddin Mohammed was responsible for writing—original draft and validation; Muhammad Abdul Mannan contributed to writing—review and editing, and data curation; Waleed Salem provided supervision.

## Consent statement

Informed written consent was obtained from the patient for the publication of this article after anonymizing the personal details.

## Declaration of generative AI and AI-assisted technologies in the writing process

During the preparation of this work, the authors used ChatGPT (OpenAI) to paraphrase some of the text. After using this tool, the authors reviewed and edited the content as needed and take full responsibility for the final publication.

## Declaration of Competing Interest

The authors declare that they have no known competing financial interests or personal relationships that could have appeared to influence the work reported in this paper.

## Data Availability

No data was used for the research described in the article.
